# Hypoxia impairs blood glucose homeostasis in naked mole-rat adult subordinates but not queens

**DOI:** 10.1242/jeb.247537

**Published:** 2024-05-21

**Authors:** Mohammad Ojaghi, Matthew E. Pamenter

**Affiliations:** ^1^ Department of Biology, University of Ottawa, Ottawa, ON, Canada, K1N 9A7; ^2^ University of Ottawa Brain and Mind Research Institute, Ottawa, ON, Canada, K1H 8M5

**Keywords:** Insulin, Development, Juvenile, Pup, Breeder

## Abstract

Naked mole-rats (NMRs) are among the most hypoxia-tolerant mammals and metabolize only carbohydrates in hypoxia. Glucose is the primary building block of dietary carbohydrates, but how blood glucose is regulated during hypoxia has not been explored in NMRs. We hypothesized that NMRs mobilize glucose stores to support anaerobic energy metabolism in hypoxia. To test this, we treated newborn, juvenile and adult (subordinate and queen) NMRs in normoxia (21% O_2_) or hypoxia (7, 5 or 3% O_2_), while measuring metabolic rate, body temperature and blood [glucose]. We also challenged animals with glucose, insulin or insulin-like growth factor-1 (IGF-1) injections and measured the rate of glucose clearance in normoxia and hypoxia. We found that: (1) blood [glucose] increases in moderate hypoxia in queens and pups, but only in severe hypoxia in adult subordinates and juveniles; (2) glucose tolerance is similar between developmental stages in normoxia, but glucose clearance times are 2- to 3-fold longer in juveniles and subordinates than in queens or pups in hypoxia; and (3) reoxygenation accelerates glucose clearance in hypoxic subordinate adults. Mechanistically, (4) insulin and IGF-1 reduce blood [glucose] in subordinates in both normoxia but only IGF-1 impacts blood [glucose] in hypoxic queens. Our results indicate that insulin signaling is impaired by hypoxia in NMRs, but that queens utilize IGF-1 to overcome this limitation and effectively regulate blood glucose in hypoxia. This suggests that sexual maturation impacts blood glucose handling in hypoxic NMR queens, which may allow queens to spend longer periods of time in hypoxic nest chambers.

## Teaser

**Summary:** The impact of hypoxia on blood glucose in hypoxia-tolerant mole-rats varies depending upon ontogeny and social status.

## INTRODUCTION

Hypoxia impairs aerobic cellular respiration; therefore, hypoxic environments pose an energetic challenge for animals. Despite this, many species inhabit and thrive in such niches. Animals that live in hypoxic environments employ cellular and physiological adaptations that enhance their tolerance to low oxygen stress (Bickler and Buck, 2007; Dzal et al., 2015; Pamenter et al., 2020; Storz and Scott, 2019). For example, many hypoxia-tolerant animals suppress their metabolic rate to offset the decrease in energy turnover during hypoxia (Guppy and Withers, 1999). Among the strategies that hypoxia-tolerant animals employ to suppress their metabolic demand is a coordinated downregulation of cellular consumers of ATP (e.g. transcription, translation, ion channel permeability, etc.) (Hochachka, 1986), matched by a downregulation of energy metabolism [i.e. the tricarboxylic acid (TCA) cycle, glycolysis, β-oxidation] (Martinez et al., 2006; Solaini et al., 2010; Storey, 1997). The role of glucose in this metabolic reorganization is of interest because glucose is a common fuel substrate derived from these pathways. Specifically, glucose is primarily metabolized via glycolysis and the TCA cycle under normoxic conditions. Furthermore, the glycerol component of lipids can contribute to glucose synthesis through gluconeogenesis, although fatty acids themselves cannot be converted into glucose in mammals. In hypoxic conditions, there may be a shift towards increased reliance on anaerobic glycolysis, leading to changes in glucose utilization and lactate production (Suh et al., 2007). Thus, elucidating how glucose metabolism is impacted by hypoxia is important to advance our understanding of how metabolic reorganization helps hypoxia-tolerant animals adapt to low O_2_ conditions.

Hypoxia has variable impacts on blood glucose. For example, hypoxia increases blood [glucose] in hypoxia-tolerant newborn calves (4.8–5.9% O_2_ for 2 h; Cheng et al., 1997) and rats (12% O_2_ for 7 days; Raff et al., 1999), but decreases blood [glucose] in adult rats (12% O_2_ for 7 days; Raff et al., 1999) and mice (10% O_2_ for 4 weeks; Lee et al., 2013). These differences seem to correlate with the relative tolerance of a given animal or developmental stage to hypoxia: neonatal animals are generally more hypoxia-tolerant than their adult counterparts and exhibit an opposing change in blood [glucose] to hypoxia (Dzal et al., 2015). Unfortunately, little information about blood glucose management during hypoxia is available from hypoxia-tolerant mammals. Fortunately, many hypoxia-tolerant adults from non-mammalian species also respond to prolonged hypoxia by increasing blood [glucose]. For example, blood [glucose] increases during hypoxia in bullfrogs (*Rana catesbeiana*; Rocha and Branco, 1997), and during anoxia in some species of freshwater turtles (e.g. *Trachemys scripta*; 9 days in 0% O_2_; Bundgaard et al., 2019). However, these animals experience chronic hypoxia or anoxia in their natural environments, and the impact of short-term hypoxia on glucose handling in hypoxia-tolerant species is poorly understood.

African naked mole-rats (NMRs; *Heterocephalus glaber*) are among the most hypoxia-tolerant mammals and putatively experience intermittent hypoxia in their densely populated underground burrows (Buffenstein et al., 2022; Pamenter, 2022). NMRs exhibit eusociality, a rare trait among mammals, where a single queen and select males reproduce while other colony members serve as non-breeding (and pre-pubertal) workers and soldiers. Colonies range from 20 to 300 individuals, characterized by a strict hierarchical structure with the queen at the top and workers performing various colony maintenance roles (Clarke and Faulkes, 2001; Holmes and Goldman, 2021; Sherman et al., 2017). As they are relatively fewer in number, breeding animals are underrepresented in scientific research, and most of the information we have about adaptations to hypoxia in this species are from studies in subordinate animals. Similarly, little attention has been paid to other developmental stages, including pups and juveniles. During exposure to environmental hypoxia, subordinate NMRs reduce their metabolic rate and body temperature (*T*_b_), and switch from mostly lipid-fueled metabolism in normoxia to carbohydrate-fueled metabolism in hypoxia (Cheng et al., 2021; Chung et al., 2016; Kirby et al., 2018; Pamenter et al., 2019; Vandewint et al., 2019). Hypoxia-mediated changes in blood [glucose] may contribute to hypoxic adaptation in this species because, like in the other hypoxia-tolerant animals described above, NMR blood [glucose] increases during hypoxia (progressive hypoxia 9–3% O_2_; 1 h each) and anoxia (4–5 min) in a laboratory setting (Pamenter et al., 2019; Park et al., 2017).

Glucose homeostasis in adult mammals is primarily mediated by insulin and insulin-like growth factor-1 (IGF-1) (Niswender, 2011). Insulin release triggers the sequestration of blood glucose into skeletal muscle and adipose tissue after a meal, where it is stored for future use (Niswender, 2011). IGF-1 can also increase glucose uptake in some peripheral tissues, and exogenous injection of IGF-1 decreases blood glucose in both healthy individuals and those with insulin resistance (Boulware et al., 1994; Schmid et al., 2005). Although the insulin receptor may mediate this response, a previous study in insulin receptor KO mice has demonstrated that the hypoglycemic effect of IGF-1 is likely mediated by IGF-1 receptors (Di Cola et al., 1997), suggesting that this hormone likely has multiple cellular targets. Although these hormones are crucial, it is important to also acknowledge the role of other regulators of glucose metabolism, including catecholamines, glucocorticoids and glucagon. Catecholamines, released during stress, play a key role in stress-induced glycogenolysis and gluconeogenesis, influencing blood glucose levels (Barth et al., 2007). Glucocorticoids, such as cortisol, contribute to increasing blood glucose by promoting gluconeogenesis and reducing glucose uptake (Sapolsky et al., 2000). Glucagon, from pancreatic alpha cells, is essential for elevating blood glucose through promoting glycogen breakdown and gluconeogenesis (Quesada et al., 2008). These regulators work in concert with insulin and IGF-1 to maintain blood glucose levels within a narrow range.

The regulation of blood glucose homeostasis in NMRs is controversial because the response of NMRs to glucose or insulin injections is different than that of mice, and because the existing literature is contradictory. For example, subordinate NMR blood [glucose] increases 15 min after glucose injection and reaches a peak value (>200 mg dl^−1^) 45±12 min post-injection, before returning to the baseline 156±31 min later (Kramer and Buffenstein, 2004). Conversely in mice, blood [glucose] reaches a greater peak value (350 mg dl^−1^) more rapidly (15 min post-injection) and returns to baseline sooner (∼1 h). These results suggest that NMRs are insulin resistant relative to mice. Conversely, when insulin is injected into NMRs, blood [glucose] drops markedly, and remains low (∼1.7 mmol l^−1^) for a prolonged time, whereas changes are slower and sustained for longer in mice, such that blood [glucose] returns to baseline after 1 h (Kramer and Buffenstein, 2004). These rapid changes in NMR blood [glucose] suggest that NMRs are more sensitive to insulin than mice. Clearly, further study is needed to resolve this confusion and better understand blood glucose regulation in NMRs during normoxia, whereas nothing is known about the regulation of glucose in NMRs during hypoxia.

To address these gaps, we explored the impact of hypoxia on blood glucose regulation and the roles of insulin and IGF-1 signaling in this regulation in NMRs throughout development. We hypothesized that NMRs rapidly mobilize glucose to help maintain anaerobic carbohydrate metabolism in hypoxia. We predicted that this response is similar across developmental stages, indicating retention of neotenic traits into adulthood (Harris et al., 2004). To test our hypothesis, we exposed NMR pups (∼2–10 days old), juveniles (∼8 weeks old), and adult subordinates (∼1–13 years old) and queens (∼3–12 years old) to 1 h of normoxia (21% O_2_), followed by 1 h of hypoxia (7%, 5% or 3% O_2_), while indirectly measuring metabolic rate via respirometry. Blood glucose and *T*_b_ were also measured. In other experiments, we challenged NMRs with bolus injections of glucose, insulin or IGF-1 in normoxia or hypoxia to evaluate the underlying mechanisms regulating blood glucose homeostasis and how these are impacted by environmental O_2_ availability in a hypoxia-tolerant mammal.

## MATERIALS AND METHODS

### Animals and ethics

All experiments were approved by the University of Ottawa Animal Care Committee and conducted in accordance with the Animals for Research Act and by the Canadian Council on Animal Care. Naked mole-rats (*Heterocephalus glaber* Rüppell 1842) were bred in the animal care facility at the University of Ottawa and housed in groups in multi-cage systems maintained at 21% O_2_, 30°C, 70% humidity and a 12 h:12 h dim light:dark cycle. Animals were fed fresh tubers, vegetables, fruits and Pronutro cereal supplement *ad libitum*. Animals were not fasted before experimental trials. A total of 229 NMRs were used in this study, including 55 adult subordinates (1–13 years old; 55±10 g), 8 queens that had previously had successful litters but that were not pregnant or lactating (3–12 years old; 65±8 g), 7 juveniles (∼2 months old; 13±2 g) and 159 pups (2–10 days old; 2.5±0.5 g). We maintained an equal representation of both sexes for the subordinate animals, but we were not able to determine sexes of pups and juveniles. For the queen and juvenile groups, the same animals were used in all experiments owing to a limited number of available animals at these developmental stages. Animals were allowed a minimum of a 2-week interval between repeated measurements to allow for adequate recovery. For subordinates, different animals were used for each experiment.

### Whole-body respirometry

A 450 ml plexiglass chamber was set inside a larger, thermally controlled chamber, which was in turn held at ∼28°C. Animals were placed individually inside of the smaller chamber and their *T*_b_ was recorded every 10 min using previously implanted RFID microchips (840 Lifechip 134 KHZ BT Domestic Equine, Destron Fearing, Langeskov, Denmark) and an RFID microchip reader (Allflex Inc., Dallas, TX, USA). To reach the desired O_2_ level, the animal chamber was sealed and ventilated with gas mixtures set by calibrated rotameters (Krohne, Duisburg, Germany). This experimental paradigm is based on extensive pilot studies with longer exposures in hypoxia (Ilacqua et al., 2017; Pamenter et al., 2019). The flow rate (*f*_I_) of gas into the animal chamber was set at 100 ml min^−1^ using a calibrated mass flow meter (Q-G265, Qubit Systems, Kingston, ON, Canada). Pups were too small to effectively measure metabolic rate in this system, or to implant RFID microchips, and so were not included in whole-body respirometry experiments.

Analyzers were calibrated before each experiment using premixed gases containing 20.95% O_2_ and 1.5% CO_2_, or 100% N_2_. Respirometry data were recorded by a Q-S102 O_2_ analyzer and a Q-S153 CO_2_ analyzer (Qubit Systems). To record the rates of oxygen consumption (*V̇*_O_2__) and carbon dioxide production (*V̇*_CO_2__), the outflow and the inflow gas concentrations were compared. To calculate *V̇*_O_2__ (ml min^−1^ kg^−1^), eqn 10.6 in Lighton (2008) was used:
(1)

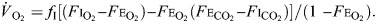
*V̇*_CO_2__ was calculated using eqn 10.7 from Lighton (2008):
(2)

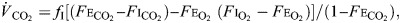
where *f*_I_ is the incurrent flow rate (ml min^−1^), *F*i_O_2__ and *F*i_CO_2__ are the fractional concentrations of incurrent O_2_ and CO_2_ of dry gas, respectively, and *F*e_O_2__ and *F*e_CO_2__ are the fractional concentrations of excurrent O_2_ and CO_2_ from the experimental chamber, respectively (Lighton, 2008). Respiratory exchange ratios (RERs) were also calculated by dividing *V̇*_CO_2__ by *V̇*_O_2__.

### Blood sampling and glucose measurements

Animals were not anesthetized before blood collection. Blood (30 μl) was collected from juvenile or adult NMRs by pricking pedal veins with a 25-gauge hypodermic needle. Blood (30–50 μl) was collected from pups by decapitation using very sharp scissors. The blood glucose concentration was then measured using a blood glucose meter (Contour next one, ASCENSIA, Mississauga, ON, Canada), which has been previously used to assay blood glucose in this species (Kramer and Buffenstein, 2004).

### Glucose, insulin and IGF-1 tests

Glucose tolerance tests were performed to determine the rate at which animals mobilize systemic responses to a glucose-load injection. We employed an insulin syringe for all injections throughout this study. Briefly, the resting blood [glucose] was measured as described above and then 2 g of glucose (dissolved in distilled water) per kilogram body mass was injected intraperitoneally (IP). For adults, the concentration of the glucose solution was 0.4 g ml^−1^, with the injection volume ranging between 0.01 and 0.05 ml, depending on the body mass. In contrast, for pups, the glucose solution concentration was set to 0.2 g ml^−1^, with the injection volume varying between 0.0015 and 0.003 ml [volume (ml)=NMR mass (kg)×dosage(g kg^−1^)/concentration (g ml^−1^)]. Next, for subordinates, queens and juveniles, the blood glucose concentration was measured at 15, 30, 45, 60, 90, 120, 150 and 180 min after glucose injection. Pups were too small to permit repeated blood draws and so individual pups were used to measure blood glucose at each time point. Importantly, because we used different pups for blood glucose measurements at each time point, the values for total glucose presented for the pup group represent an estimation for this developmental stage because it was not possible to conduct repeated sampling in the same individuals. For glucose tolerance tests in hypoxia, animals were first exposed to a hypoxic environment for 1 h, with baseline blood glucose measurements taken before and after hypoxia. Animals then received a glucose injection and were returned to the hypoxic chamber rapidly after each blood sample. Next, blood glucose levels were measured at 30, 60, 90, 120, 150 and 180 min after injection in hypoxia.

The same procedure was used for both the insulin and IGF-1 tolerance tests. Insulin was diluted 1:1000 in 0.9% NaCl and then was injected IP (0.75 U insulin kg^−1^ body mass; human insulin, Gibco, India, 4 mg ml^−1^; stock: 100 U ml^−1^ insulin; working concentration 0.1 U ml^−1^ for adults and 0.05 U ml^−1^ for pups). Similarly, human IGF-I (Shenandoah Biotechnology, USA; 1 mg kg^−1^ body mass; working concentration 0.3 mg ml^−1^) was injected IP to queens and subordinates. After injections or glucose measurements, animals were promptly returned to the chamber, typically within 3 min, and the chamber was resealed. We maintained a high flow rate in the chamber during tolerance tests compared with metabolic experiments to ensure a quick return to hypoxia, and the chamber oxygen level was re-established within 5 min of returning the animal (data not shown).

### Statistical analysis

Logger Pro 3 software (Vernier Software & Technology, Beaverton, OR, USA) was used to record the incurrent and the excurrent O_2_, CO_2_ and *f*_I_ levels. We calculated the average of *T*_b_, *V̇*_O_2__ and *V̇*_CO_2__ using the last 10–15 min of each O_2_ exposure (21%, 7%, 5% and 3% O_2_). Statistical analysis was performed using GraphPad Prism 9 (GraphPad Prism, La Jolla, CA, USA). All data followed a normal distribution with equal variance (*P*>0.05). Significant differences (*P*<0.05) were determined with one-way ANOVAs and mixed-effects model analysis (REML) to examine intraspecific differences for each independent variable: (1) normoxia and (2) O_2_ level (7%, 5% or 3% O_2_) or (3) glucose, insulin or IGF-1 injection. When a significance difference was determined, Tukey's or Šidák's multiple comparisons tests were used to identify significantly different normoxia within individual treatment groups. Data are presented as means±s.e.m., where *P*<0.05 was the threshold for significance.

## RESULTS

### The hypoxic metabolic response is similar across developmental stages

**Fig. 1.**
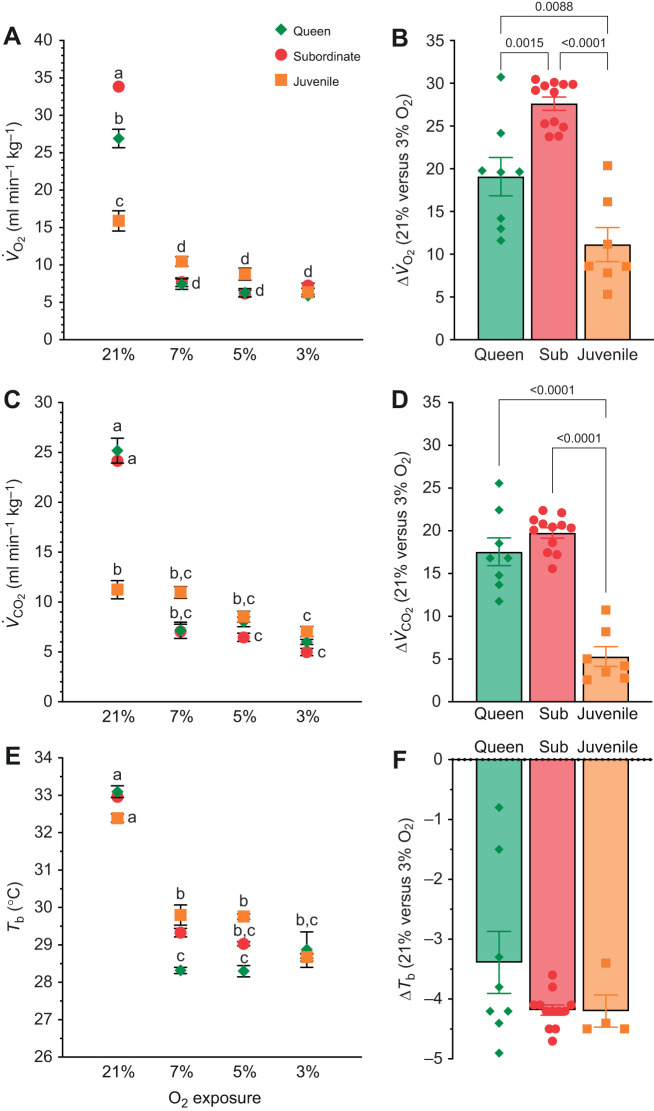
**Naked mole-rats (NMRs) decrease metabolic rate and body temperature in all hypoxic levels and developmental stages.** (A) Oxygen consumption (*V̇*_O_2__) from subordinate (*n=*7–12), queen (*n=*5–8) and juvenile (*n=*4–5) NMRs held for 1 h in normoxia (21% O_2_), then exposed for 1 h in acute hypoxia (7, 5 or 3% O_2_). (B) Change (Δ) in *V̇*_O_2__ from animals treated in A. (C) Carbon dioxide production (*V̇*_CO_2__) from animals in A. (D) Δ*V̇*_CO_2__ for data shown in C. (E) Body temperature (*T*_b_) for animals treated in A. (F) Δ*T*_b_ for data shown in E. Data are presented as means±s.e.m. Significant differences are indicated by different letters (two-way ANOVA, *P<*0.05).

We first evaluated metabolic responses to three different levels of acute hypoxia in juvenile, subordinate and queen NMRs (Fig. 1; note that the small size and sensitivity to isolation of pups prevented us from measuring metabolic responses in this developmental stage). In normoxia, subordinates had the highest metabolic rate (as measured indirectly using respirometry; Fig. 1A), followed by queens. The normoxic metabolic rate of juvenile NMRs was ∼40–50% lower than that of the older animals (*F*_2,58_=11.7, *P<*0.0001 for *V̇*_O_2__; *F*_2,58_=6.1, *P<*0.0001 for *V̇*_CO_2__). With the onset of hypoxia, metabolic rate decreased in all animals and age groups, by ∼75–80% in subordinates, ∼70–75% in queens and ∼40–60% in juveniles (*F*_3,57_=392.7, *P*<0.0001 for *V̇*_O_2__; *F*_2,58_=286.1, *P<*0.0001 for *V̇*_CO_2__; Fig. 1A–D). There was no significant difference between metabolic rates across the three levels of hypoxia tested for any developmental stage, and all three developmental stages reached a similar minimum metabolic rate in severe hypoxia (3% O_2_). Finally, although the degree of change was significantly different between each developmental stage (Fig. 1B,D), these differences were due to variance in normoxic metabolic rates.

To evaluate the role of thermoregulation in the hypoxic hypometabolic state across development, we also measured *T*_b_ (Fig. 1E,F). Subordinates, queens and juveniles had a similar *T*_b_ in normoxia, and animals at all three developmental stages exhibited a rapid decrease in *T*_b_ with the onset of hypoxia (*F*_3,54_=545.5, *P*<0.0001). The decrease in *T*_b_ was most sensitive to hypoxia in queens, in which *T*_b_ decreased further in 7 and 5% O_2_ than in the other developmental stages. However, all three developmental stages had a similar magnitude of *T*_b_ decrease in the most severe level of hypoxia tested (3% O_2_).

We also calculated RERs to gain insight into fuel use in each condition and by animals in each developmental stage (Table 1). The RER is an indicator of fuel usage (e.g. carbohydrates or lipids): a value of 0.7 indicates lipid metabolism and a value of 1.0 indicates carbohydrate metabolism, whereas a value between 0.7 and 1 indicates a mix of both lipid and carbohydrate (Petersson and Glenny, 2014). With the onset of hypoxia, both subordinate and juvenile NMRs exhibited a shift from predominately lipids towards predominately carbohydrates (*F*_3,56_=42.57, *P*<0.0001). Queens relied primarily on carbohydrates in all experimental conditions and did not exhibit an RER shift upon the switch to hypoxia. Notably, NMRs do not hyperventilate (through increased ventilation, the typical hypoxic ventilatory response; Pamenter, 2023; Pamenter et al., 2019), and so this RER shift is not likely to be driven by a change in ventilation.

**Table 1.**
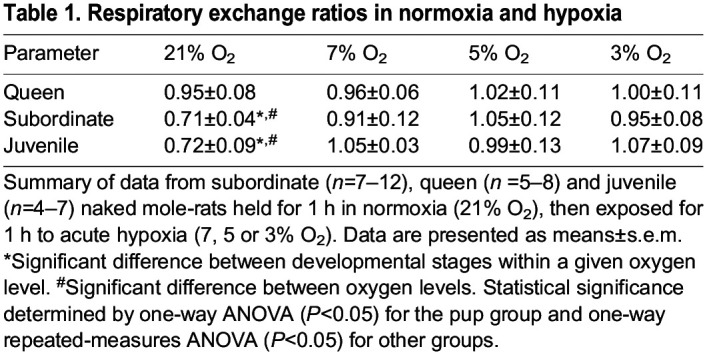
Respiratory exchange ratios in normoxia and hypoxia

### Blood glucose is differentially elevated in hypoxia between developmental stages

**Fig. 2.**
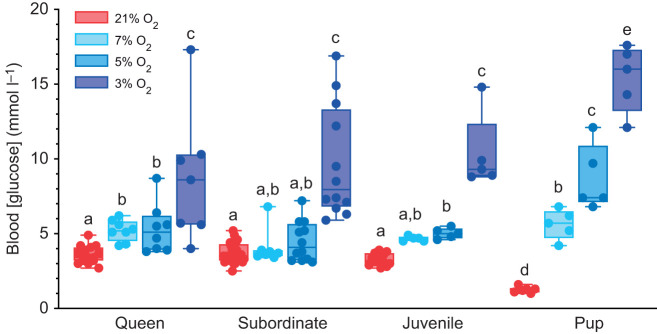
**Blood glucose increases with severe hypoxia in NMRs.** Each group of NMRs were exposed to 21% O_2_ or hypoxia (3%, 5% and 7%) for 1 h, as in Fig. 1. Data are presented as means±s.e.m. Significant differences are indicated by different letters (two-way ANOVA for the pup group and two-way repeated-measures ANOVA for other groups, *P<*0.05).

We next evaluated the impact of acute hypoxic exposure on blood [glucose] (Fig. 2). In subordinate adult, queen and juvenile NMRs, normoxic blood [glucose] was similar and blood [glucose] was only moderately or not significantly upregulated in 5 or 7% O_2_. However, blood [glucose] increased ∼2-fold above normoxic levels in all animals exposed to 3% O_2_ for 1 h (*F*_1.4,33.59_=121.5, *P*<0.0001). Conversely, the blood [glucose] of normoxic pups was significantly lower than in the other developmental stages, and exhibited a graded response to acute hypoxia exposure, such that blood [glucose] increased ∼5-, 10- and 15-fold in 7, 5 and 3% O_2_, respectively, relative to normoxic levels (*P*=0.0017, 0.0072 and 0.0005, respectively). The maximum blood [glucose] in pups was ∼50% higher than that of the other developmental stages.

### Subordinates and juveniles have a longer blood [glucose] latency than queens and pups

**Fig. 3.**
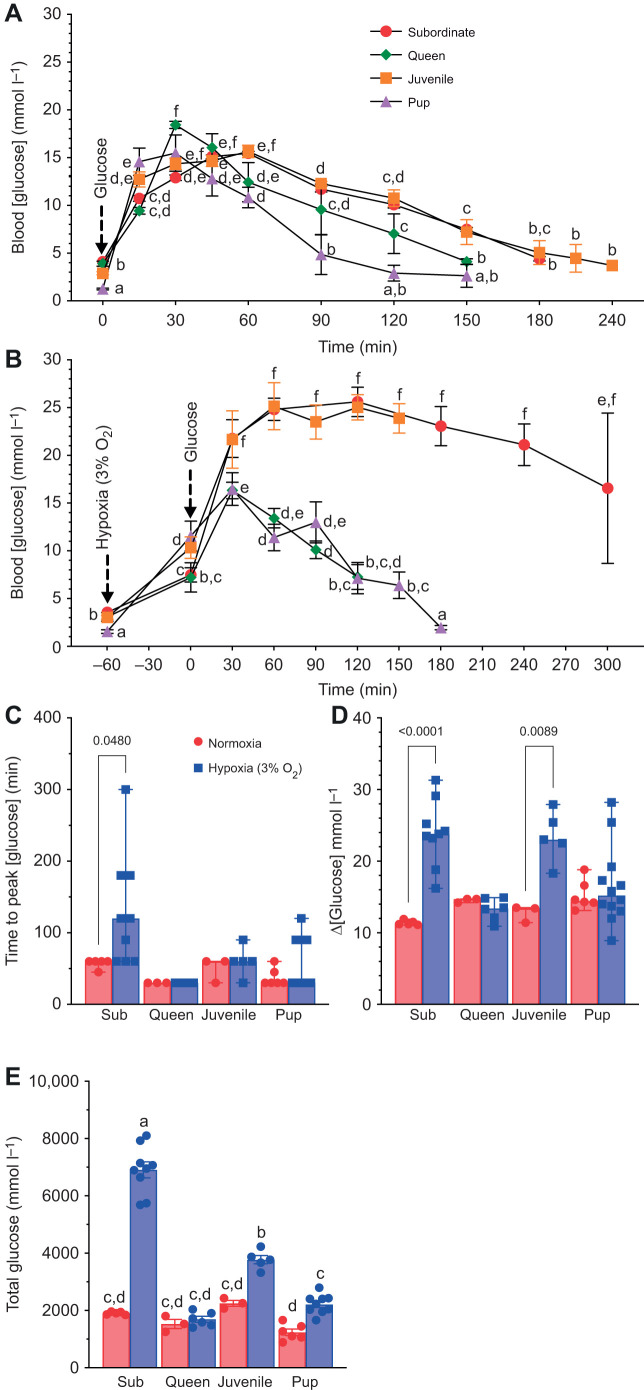
**Hypoxia increases the latency of glucose tolerance tests.** (A) Blood [glucose] before and after injection of 2 g glucose kg^−1^ body mass in normoxia (*n*=5 for subordinates, 3 each for queens and juveniles, and 37 for pups). (B) Blood [glucose] before and after injection of 2 g glucose kg^−1^ body mass in hypoxia (3% O_2_) (*n*=9 for subordinates, 6 for queens, 5 for juveniles and 51 for pups). (C) Time required to reach peak blood glucose value under normoxia and hypoxia. (D) Change (Δ) in blood glucose concentration from peak to baseline levels. (E) Total glucose (area under the curve from the baseline level to the point of return to baseline value). Data are presented as meanss±s.e.m. Arrows indicate onset of hypoxia exposure or point of glucose injection, as specified in each panel. Significant differences are indicated by different letters (two-way ANOVA for the pup group and two-way repeated-measures ANOVA for the other groups, *P<*0.05).

Next, we evaluated the ability of NMRs to re-establish blood glucose homeostasis after a bolus IP glucose challenge in normoxia or hypoxia (Fig. 3). For this comparison we utilized a hypoxia challenge of 3% O_2_ because this was the level of hypoxia at which all age groups exhibited an increase in blood [glucose]. In normoxia, the peak magnitude of blood [glucose] following the glucose challenge was similar between all developmental stages (*F*_10,128_=36.85, *P*<0.0001; Fig. 3A,D), but the time to peak blood [glucose] and the decay time representing glucose clearance rate were both greater in subordinates and juveniles (*F*_3,13_=8.73, *P*=0.002; Fig. 3A,C). Specifically, blood [glucose] reached a maximum value ∼30–40 min post-injection in pups and queens versus ∼60 min post-injection in juveniles and subordinate adults. Similarly, blood [glucose] returned to near baseline levels ∼150 min post-injection in pups and queens, but it took between 180 and 240 min for blood [glucose] to return to near baseline levels in juveniles and subordinate adults. Thus, the decay time from the peak in blood [glucose] to baseline was shorter for pups and queens than for subordinates and juveniles (Fig. 3A).

Intriguingly, hypoxia had only minor effects on peak blood [glucose], time to peak, or the time required to clear the exogenous glucose challenge in either queens or pups. Specifically, blood [glucose] reached a peak value ∼30 min post-injection in queens and pups, and blood [glucose] returned to near baseline values after ∼150 min (*F*_9,145_=20.56, *P*<0.0001; Fig. 3B–D). Conversely, hypoxia considerably increased the peak magnitude of blood [glucose] and the clearance time in both subordinate adult and juvenile NMRs (*F*_3,25_=16.24, *P*<0.0001), whereas the time to peak blood [glucose] was elevated in subordinates only. Specifically, blood [glucose] peaked and then plateaued between 60- and 120-min post-injection in these developmental stages, and at a peak magnitude that was approximately two-thirds greater than the blood [glucose] peak in animals at the same developmental stage receiving a similar injection in normoxia. Furthermore, the time to clear this bolus glucose challenge was prolonged, and blood [glucose] remained elevated above the normoxic glucose challenge peak for up to 5 h post-injection in subordinates (*F*_1,26_=13.01, *P*=0.0013), which was the limit permitted for experimentation under our animal ethics protocol. Thus, the decay time from peak blood [glucose] to baseline in hypoxia was considerably less for queens and pups than for subordinates and juveniles. We also analyzed the area under the curve as a measure of total glucose mobilized to the blood by a given treatment. Here, values were consistently higher in hypoxia than normoxia across all groups, which suggests an increased glucose response when oxygen levels are lower. In contrast, there was no difference in total glucose mobilized in queens between normoxia and hypoxia, unlike the other groups. Subordinates had the largest amount of glucose mobilized in hypoxia, whereas the pups had the lowest amount in normoxia. Additionally, there were no significant differences in total glucose among any of the groups when in normoxia (*F*_3,38_=65.1, *P*<0.0001; Fig. 3E).

### Reoxygenation accelerates the rate of glucose clearance in subordinate NMRs

**Fig. 4.**
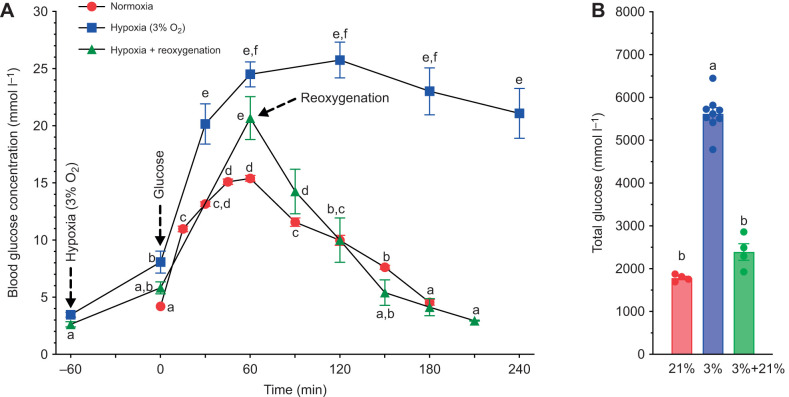
**Reoxygenation accelerates removal of glucose from the blood of subordinates NMRs.** (A) Blood [glucose] before and after injection of 2 g glucose kg^−1^ body mass in normoxia, hypoxia (3% O_2_) or hypoxia followed by reoxygenation after 1 h (*n*=4 each). (B) Total glucose (area under the curve from the baseline level to the point of return to baseline value). Arrows indicate onset or offset of hypoxia exposure and/or point of glucose injection, as specified in the figure. Significant differences are indicated by different letters (one-way repeated-measures ANOVA, *P<*0.05).

In a subset of experiments, a group of subordinate adults were re-exposed to normoxia 1 h into the glucose tolerance test protocol in hypoxia (3% O_2_; *F*_2,105_=47.95, *P*<0.0001 for hypoxia; Fig. 4A). Upon reoxygenation, blood [glucose] rapidly began to decline and returned to pre-injection normoxic baseline in a similar timeframe as in animals undergoing a glucose tolerance test in normoxia. Calculation of total glucose supports these observations, indicating that the values during reoxygenation are close to those seen in normoxic conditions (*F*_2,14_=189.2, *P*<0.0001; Fig. 4B).

### Queens and pups, but not adult subordinates, are insensitive to insulin in hypoxia

**Fig. 5.**
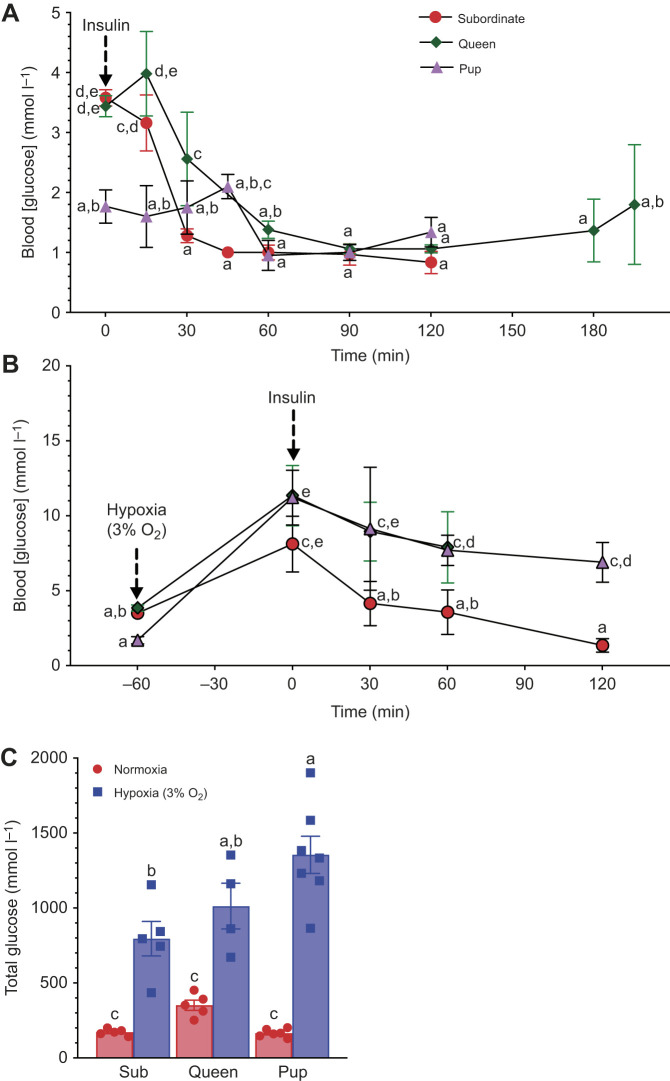
**Insulin reduces blood [glucose] in queens and subordinates but not pups.** (A) Blood [glucose] before and after injection of 0.75 U insulin kg^−1^ body mass in normoxia. (B) Blood [glucose] before and after injection of insulin in hypoxia (3% O_2_). (C) Total glucose (area under the curve from the baseline level to the point of return to baseline value). Data are presented as means±s.e.m. (*n*=5 for subordinates, 4–5 for queens and 50 for pups). Arrows indicate onset of hypoxia exposure or point of insulin injection, as specified in the figure. Significant differences are indicated by different letters (two-way ANOVA for the pup group and two-way repeated-measures ANOVA for other groups, *P<*0.05).

To elucidate the role of key hormones in the regulation of blood glucose, we next evaluated the sensitivity of NMR blood [glucose] to an insulin tolerance test (Fig. 5). In normoxia, insulin injection resulted in rapid decreases of blood [glucose] in queens and subordinate adult NMRs, such that blood [glucose] dropped significantly 30 min following insulin injection (*F*_8,40_=10.5, *P*<0.0001; Fig. 5A). Conversely, insulin injection had no effect on blood [glucose] in pups in normoxia. In hypoxia, insulin similarly reduced blood [glucose] in adult subordinates and had no effect on pups; however, insulin injection also had no impact on blood [glucose] in hypoxic queens (*F*_4,24_=4.38, *P*=0.0084; Fig. 5B), suggesting that these animals were not sensitive to insulin in hypoxia. Analysis of total glucose indicates that considerably more glucose is mobilized in hypoxia than in normoxia across all groups. Pups in hypoxia displayed the largest glucose mobilization values, whereas subordinates and pups in normoxia had minimal total glucose values (*F*_2,26_=6.2, *P*=0.0063; Fig. 5C).

### Hypoxic blood [glucose] changes are IGF-1 sensitive in adults

**Fig. 6.**
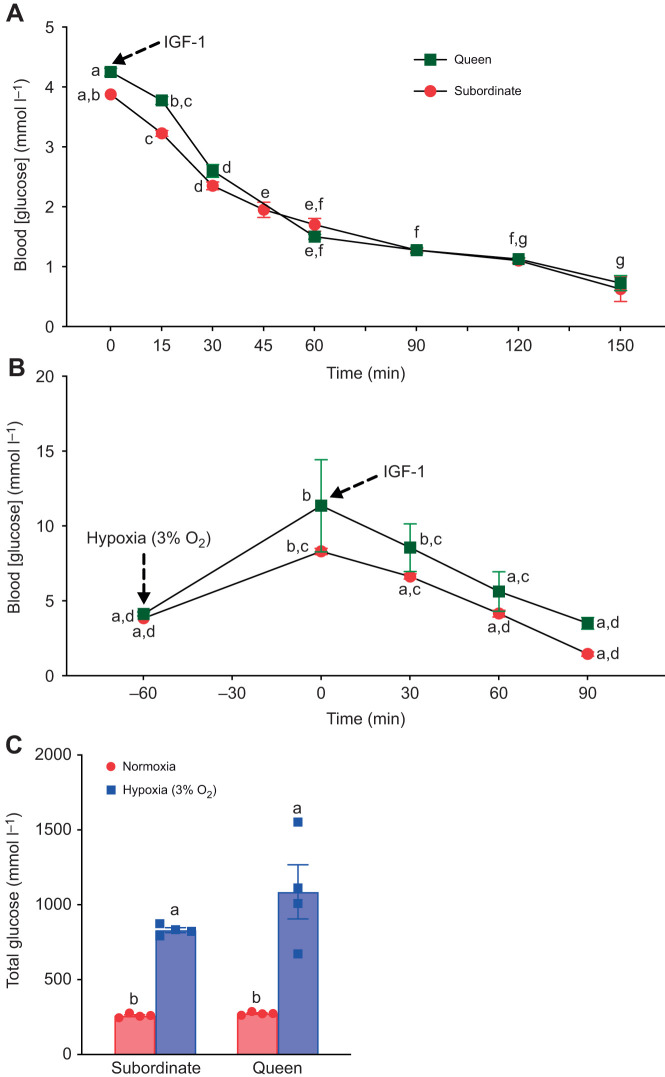
**IGF-1 reduces blood [glucose] in queens and subordinates.** (A) Blood [glucose] before and after injection of IGF-1 (1 mg kg^−1^ body mass) in normoxia. (B) Blood [glucose] before and after injection of IGF-1 in hypoxia (3% O_2_). (C) Total glucose (area under the curve from the baseline level to the point of return to baseline value). Data are presented as means±s.e.m. from *n*=4 each. Arrows indicate onset of hypoxia exposure or point of IGF-1 injection, as specified in the figure. Significant differences are indicated by different letters (two-way repeated-measures ANOVA, *P<*0.05).

Finally, we evaluated the impact of IGF-1 injection on blood [glucose] in adult subordinates and queens (Fig. 6). Here, IGF-1 tolerance tests during normoxia demonstrated that both queen and subordinate adult NMRs are sensitive to IGF-1 (*F*_7,21_=264.1, *P*<0.0001; Fig. 6A). Similarly, in hypoxia, IGF-1 injection decreased blood [glucose] in both subordinate adults and queens (*F*_4,12_=20.6, *P*<0.0001; Fig. 6B). Interestingly, the changes in blood glucose in normoxia were slower than in hypoxia. Specifically, the blood [glucose] of normoxic animals reached the lowest level at 120±15 min post-IGF-1 injection, whereas during hypoxia, it was at 75±15 min post-IGF-1 injection. Analysis of total glucose mobilization revealed significant differences between normoxic and hypoxic conditions across all groups. Notably, under hypoxic conditions, total glucose mobilized in queens was higher than that for subordinates, suggesting that the queen group may experience a substantially greater overall blood glucose responses compared with the subordinate group (*F*_1,12_=1.74, *P*=0.211; Fig. 6C).

## DISCUSSION

Glucose is a key metabolic intermediate and is often released into the blood during hypoxia to support anaerobic metabolism. As such, differences in the regulation of blood glucose during hypoxia may be an important area of adaptation in hypoxia-tolerant species. In the present study, we evaluated metabolism, *T*_b_ and glucose handling during normoxia and hypoxia and across development in NMRs, which are among the most hypoxia-tolerant mammals (Buffenstein et al., 2022; Pamenter, 2022). Our study yielded several important findings. First, we describe metabolic and thermoregulatory responses to acute hypoxia across development for the first time in NMRs and report that animals of all developmental stages exhibited robust decreases in metabolic rate and thermogenesis in all levels of hypoxia tested. Conversely, blood glucose homeostasis is less sensitive to hypoxia than metabolism; juveniles and subordinates did not exhibit increased blood [glucose] until the most severe level of hypoxia tested whereas pups and queens exhibited increased blood [glucose] in moderate hypoxia and graded increases in blood [glucose] with progressively more severe hypoxia. Despite their high activation threshold for glucose mobilization by hypoxia, subordinates and juveniles had a higher blood [glucose] peak and took hours longer to clear a bolus glucose injection from the blood than pups or queens, suggesting that glucose handling is impaired by hypoxia in this species. Remarkably, glucose clearance accelerated to match the normoxic clearance rate if subordinate animals were returned to normoxia, confirming that the cause of this impairment is oxygen-sensitive, at least at this developmental stage. Presumably, this impairment is at the level of insulin release because exogenous insulin induced the removal of glucose from the blood in all developmental stages and experimental conditions, indicating that the insulin receptor remains functional. Conversely, queens and pups had reduced sensitivity to insulin during hypoxia, suggesting that insulin receptor function is modulated by hypoxia in sexually developed females. However, these animals remained sensitive to IGF-1, which presumably allows them to maintain active regulation of blood glucose in hypoxia. Together, these results indicate that (1) glucose mobilization is not a primary response to reduced metabolic demand during low oxygen availability and is only recruited in severe hypoxia in most NMRs, and (2) the signaling pathways that regulate glucose are variably modified by hypoxia, depending on developmental and/or reproductive stage.

### NMRs of all developmental stages reduce metabolic demand and thermogenesis in hypoxia

Previous studies overwhelmingly agree that, in response to acute hypoxia exposure, subordinate adult NMRs exhibit a robust hypoxic metabolic response characterized by a decrease in metabolic rate of up to 85%, a cessation of non-shivering thermogenesis that allows *T*_b_ to drop to near ambient temperatures, and a fuel substrate switch from mixed lipids and carbohydrates to entirely carbohydrates (Cheng et al., 2021; Chung et al., 2016; Dzal et al., 2019; Farhat et al., 2020; Houlahan et al., 2018; Ilacqua et al., 2017; Kirby et al., 2018; Pamenter, 2022; Pamenter et al., 2015, 2019, 2018; Vandewint et al., 2019). In the present study, we extend these findings to juveniles and breeding female NMRs (queens) and demonstrate that, like adult subordinates, these developmental groups also reduce *V̇*_O_2__ and *V̇*_CO_2__ progressively with increasingly deeper levels of hypoxia, reduce *T*_b_ to near ambient levels, and rely on carbohydrate metabolism in hypoxia. Conversely, most other small mammals, including rats, mice, hamsters and ground squirrels, utilize divergent physiological strategies for coping with hypoxia depending on their stage of development (Dzal and Milsom, 2019, 2021; Frappell et al., 1992; Mortola et al., 1989). For example, most other adult rodents exhibit a lesser depression in metabolic rate under hypoxic conditions compared with neonates or juveniles of the same species, and this correlates with diminished hypoxia tolerance in adults relative to neonates.

Regardless of developmental stage, a switch in primary metabolic fuel substrate usage from lipid to carbohydrates during hypoxia provides a greater yield of ATP per mole of O_2_ consumed, which can increase energy efficiency, and is often observed in small hypoxia-tolerant mammals (Ivy et al., 2020; Schippers et al., 2012). In NMRs, this shift is supported by the mobilization of glucose stores from the liver and an increase in blood glucose levels during severe (Pamenter et al., 2019), but not moderate hypoxia (present study, see below). Combined with a significant suppression of metabolic rate, this mobilization may help to limit the need for anaerobic metabolism and reduce the subsequent oxygen debt that occurs after severe hypoxia.

### Adult and juvenile NMRs mobilize glucose only in severe hypoxia

In general, most hypoxia-intolerant species have variable glucose responses to hypoxia, which are often characterized by reductions in blood [glucose], whereas hypoxia-tolerant species exhibit elevated blood [glucose] in hypoxia or anoxia (see Introduction and Bundgaard et al., 2019; Cheng et al., 1997; Lee et al., 2013; Raff et al., 1999; Rocha and Branco, 1997). In good agreement with the pattern presented by these studies, we report increased blood glucose during hypoxia in all developmental stages of NMRs. This response was most sensitive in queens and pups, which exhibited increased blood [glucose] in 7% O_2_. Conversely, juveniles and subordinates did not exhibit increases until 5 and 3% O_2_, respectively. Where present, changes in blood glucose induced by 7 and 5% O_2_ were mild, but then jumped markedly in 3% O_2_. Taken together, these data demonstrate that NMRs at most developmental stages require a very strong hypoxic stimulus to mobilize glucose into the blood. This is important because elevations of glucose in blood may occur because of a release of stored glucose from tissue (primarily liver) into the blood and/or a reduction in glucose uptake by metabolizing tissues themselves, such as occurs during periods of metabolic rate suppression. Importantly, the suppression of metabolic rate that we observed in all developmental stages in 7% O_2_ is near the maximum suppression observed in deeper levels of hypoxia, but 7% O_2_ is a level of hypoxia that is well above the threshold required for significant glucose accumulation in the blood of these animals. Therefore, the hypoxic increase in blood [glucose] is likely not mediated by a decrease in tissue uptake. In support of this, we have previously demonstrated that the expression of GLUT-4 (which is the primary mediator of glucose uptake into cells) and phosphorylated AMPK (which activates glucose uptake and glycolysis) are downregulated in total NMR skeletal muscle during moderate hypoxia (7% O_2_ for 4 h; Hadj-Moussa et al., 2021), which would contribute to reduced glucose uptake by that tissue. Conversely, in 3% O_2_, blood [glucose] increases sharply in all developmental stages and liver [glucose] plummets (Pamenter et al., 2019), suggesting that glucose stores are mobilized from the liver only in severe hypoxia, presumably to support increased anaerobic activity in tissues.

### Divergent regulation of blood [glucose] in hypoxia

Glucose tolerance tests indicate that NMRs are glucose tolerant across all developmental stages in normoxia (Edrey et al., 2011). Conversely, we report starkly different responses to glucose injection in hypoxia across development, such that queens and pups retain glucose tolerance in hypoxia, whereas juveniles and subordinate adults are less able to effectively clear the exogenous glucose bolus from their blood, suggesting that the signaling mechanisms responsible for mediating this removal are impaired in hypoxia. These differences are clearly O_2_-sensitive because when subordinate adult NMRs are reoxygenated following 1 h of hypoxia post-injection, blood [glucose] rapidly declined to baseline values.

Insulin is the primary initiator of glucose uptake in most mammals, and insulin signaling is upregulated by hypoxia in hypoxia-tolerant newborn calves (Cheng et al., 1997) and juvenile and newborn rats (Raff et al., 1999), but decreases in hypoxia-intolerant adult rats (Chen et al., 2007). Unfortunately, we were unable to measure insulin levels in NMRs because commercial insulin kits do not effectively detect insulin in this species (Kramer and Buffenstein, 2004), presumably because NMRs (along with other hystricognaths) have mutations in their insulin β-chain sequence (Fang et al., 2014), which are consistent with reduced affinity for insulin receptors (Horuk et al., 1980; Liu et al., 2010). Nonetheless, and consistent with a previous study in NMRs (Kramer and Buffenstein, 2004), insulin tolerance tests in normoxia demonstrate that NMRs are responsive to insulin. Furthermore, similar tests in hypoxia indicate that subordinates, but not queens and pups, remain sensitive to insulin when O_2_ is limited. These data suggest a potential developmental effect on the hypoxic regulation of insulin signaling in NMRs such that insulin receptors are impaired by hypoxia in reproductive queens and pups, but not subordinate adults or juveniles. Conversely, the inability of subordinate adults and juveniles to clear an exogenous glucose challenge suggests that glucose sensing and/or insulin release may be impaired by hypoxia in these developmental stages, because glucose uptake remains responsive to exogenous insulin in hypoxia.

Sex hormones have marked impacts on insulin signaling; therefore, it is possible that queens develop a response that is different from that of subordinate animals as they undergo sexual differentiation. For example, estrogen plays an important role in regulating insulin sensitivity and the expression of gluconeogenesis enzymes (Bian et al., 2019; Yan et al., 2019). Specifically, estrogen indirectly inhibits Foxo1, which is a regulator transcription factor for hepatic glucose production and promotes the gene expression of gluconeogenesis enzymes via activation of PI3K-AKT signaling (Guo, 2014; Zhao et al., 2004). Estrogen levels are higher in NMR queens than in subordinates (Edwards et al., 2021), which may explain some of these developmental effects. Furthermore, the hormonal control of glucose regulation in pups may be like that in queens if pups ingest estrogen from the mother while nursing. Then, as animals are weaned and develop into sexually supressed subordinate adults, their estrogen levels would fall concomitant with changing insulin sensitivity. Further study is needed to investigate how estrogen may impact blood glucose and insulin signaling in this species.

It is also notable that in most mammals, the insulin signaling system is not functional until post-birth; instead, insulin-like growth factors regulate blood [glucose] through insulin receptors at this developmental stage (Liang et al., 2010). Post-birth, the IGF system declines, and insulin signaling takes over in most mammals (Lui and Baron, 2013). The IGF system of NMRs is functionally similar to that of humans and mice (Brohus et al., 2015), indicating that there may not be significant adaptations in this signaling pathway in NMRs per se; however, the expression of IGF and its receptors persists into adulthood in NMRs (Fang et al., 2014), unlike in most other mammals. Together with the mutations to the insulin sequence discussed above, these data suggest that, unlike most adult mammals, NMRs may retain a fetal IGF signaling pathway into adulthood. Of particular interest is IGF-1, which indirectly enhances glucose uptake by promoting the expression of glucose transporters, such as GLUT 4 (Mora et al., 1995). IGF-1 can also promote the activity of enzymes involved in glucose metabolism, such as hexokinase and pyruvate dehydrogenase, thereby enhancing the utilization of glucose for ATP production in cells (Kasprzak, 2021). We found that both queen and subordinate NMRs are sensitive to IGF-1 injection during both normoxia and hypoxia, suggesting that IGF signaling indeed plays a crucial role in regulating glucose metabolism in NMRs and may therefore provide queens with a mechanism to regulate blood [glucose] in hypoxia when they are insensitive to insulin signaling.

### Study limitations

A key limitation of our study is the absence of a sham control group; repeated animal handling during the administration of injections, and the collection of blood without the use of anesthetic, may have induced stress. Such stress could lead to elevations of catecholamines and glucocorticoids, which can, in turn, affect blood glucose levels. In this study, our objective was to compare changes across different oxygen level groups. Consequently, we ensured that all groups were subjected to identical handling procedures if possible, and thereby standardizing the stress from handling across most experimental conditions (exclusing pups, which were sampled a single time each). We believe that this design choice, along with our consideration of the physiological effects of stress on blood glucose levels, supports a valid comparison of the impact of hypoxia on blood glucose across development. However, this cavest should be kept in mind when evaluating our results.

### Conclusions

Our study provides insight into the importance of glucose in the reorganization of NMR metabolism during hypoxia, and the function of insulin and IGF-1 signaling in this regulation. An intriguing conclusion that may be drawn when considering the ecophysiological significance of our results is that NMRs likely do not experience the severe depths of hypoxia in their natural habitats that are required to induce glucose mobilization in our study (Buffenstein et al., 2022; Holtze et al., 2018; Pamenter, 2022, 2023). This is important because it suggests that NMRs do not need to release glucose stores into the blood to support anaerobic metabolism except when approaching the limits of their hypoxia tolerance (Pamenter et al., 2015, 2019). Presumably, this indicates that NMRs can effectively and coordinately downregulate energy demand to match declining energy supplies in moderate hypoxia at all stages of development. Furthermore, queens, as the reproductive and dominant individuals in the colony, appear to have evolved a strategy to regulate their blood [glucose] efficiently, even in severe hypoxia, when insulin-mediated glucose regulation appears to be otherwise disabled in adult NMRs. NMRs putatively experience hypoxia primarily in their nest chambers, and queens remain in these chambers far longer than other conspecifics while birthing, feeding and caring for pups (Buffenstein et al., 2022; Pamenter, 2022), which would also require significant energy. Therefore, queens likely have the most regular and intense exposure to hypoxia in the colony, but also have the highest metabolic demands due to reproductive costs. As such, reproductive females may require anaerobic energy production more frequently or in less severe levels of hypoxia than do subordinate adults or juveniles, and thus need to retain the ability to recruit glucose reserves to support tissue-level carbohydrate metabolism during periods of hypometabolism in hypoxia. Alternatively, queens may need to reserve lipids to produce milk or as building blocks for growing fetuses, and thus require a more ready and rapid pool of carbohydrate substrate in all conditions. In either case, this framework is supported by our observation that blood [glucose] becomes elevated in queens at a less severe level of hypoxia than in subordinate animals. Further research into the mechanisms underlying IGF-1 regulation and potential interactions between estrogen and glucose homeostasis in NMRs is clearly warranted to better understand the metabolic adaptations of this species and their potential relevance to human health.
